# The prevalance of binge eating disorder and associated psychiatric and substance use disorders in a student population in Kenya – towards a public health approach

**DOI:** 10.1186/s12888-022-03761-1

**Published:** 2022-02-16

**Authors:** Victoria N. Mutiso, David M. Ndetei, Esther N Muia, Rita K Alietsi, Lydia Onsinyo, Frida Kameti, Monicah Masake, Christine Musyimi, Daniel Mamah

**Affiliations:** 1 Africa Mental Health Research and Training Foundation, Mawensi Road, Off Elgon road, Mawensi Garden, P.O. Box 48423-00100, Nairobi, Kenya; 2 Department of Psychiatry, University of Nairobi, Mawensi Road, Off Elgon road, Mawensi Garden, P.O. Box 48423-00100, Nairobi, Kenya; 3 Department of Public and Community Health, Machakos University, Machakos, Kenya; 4 Department of Psychiatry, Washington University Medical School, St. Louis, Missouri USA

**Keywords:** Eating Disorders, Binge Eating, Co-morbidity, Kenya

## Abstract

**Introduction:**

Kenya in particular and Africa in general lack data on Binge Eating Disorder (BED). The overarching objective of this study is to fill that gap. Kenyans may not be aware that BED exists when a “very good” appetite is considered a sign of good health, especially if food is available either at home, in fast food shops or when communally eating together, a very common cultural practice. On the other hand where there is relatively insufficient food, it is not expected that one could be having a problem of eating too much.

**Method:**

We administered the following tools and measurements to 9742 participants (high school, college and university students): 1) Researcher designed socio-demographic and economic indicator questionnaire; 2) An instrument documenting DSM-IV diagnostic criteria for BED and its various symptoms; 3) An instrument to determine DSM-IV psychiatric disorders and substance abuse;4) An instrument measuring high risk for psychosis ,affectivity and stress; 5) A WHO designed instrument measuring the severity of substance abuse for specific substances. We used descriptive and inferential analysis to determine the prevalence and association of the different variables. Independent predictors of BED were generated from a generalized linear model (*p*<0.05).

**Results:**

We found a prevalence of 3.2% of BED and a wide range of prevalence for BED and BED related symptoms (8.1% to 19%). The least prevalent was "To prevent weight gain from eating binge did you force yourself to vomit, or used laxatives?”. The most common was "Did you often go on eating binges (eating a very large amount of food very quickly over a short period of time)." Major depression, obsessive compulsive disorder, panic disorder, agoraphobia, generalized anxiety disorder ,a positive stress screen and drug abuse were independent predictors of BED (*p*<0.05).

**Conclusion:**

Our findings on the prevalence of BED and significant associations with various psychiatric disorders and substance use disorders are similar to those obtained in High Income Countries (HIC) using similar large-scale samples in non-clinical populations. Our findings suggest the need fora public health approach to enhance awareness of BED and to promote health-seeking behaviour towards management of BED.

## Introduction

BED entails “the occurrence of binge episodes marked by consumption of large quantities of food without control in the absence of the extreme weight-control behaviours characteristic of bulimia nervosa” [1, 2]. The commonest of eating disorders is BED [3, 4].

### Prevalence of binge eating disorder

Studies using DSM-V have generally found higher prevalence of BED compared with studies using DSM-IV, for example, an Australian study found a 50% increase of DSM-V prevalence of BED over DSM-IV from same cohorts [5]. In Europe, prevalence of BED using DSM-V is higher by 1-4% compared with DSM-IV [6].

### Co-morbidity with psychiatric disorder

Most studies on the co-morbidity of psychiatric disorders and eating disorders have not given a breakdown on which particular psychiatric disorders are associated with which types of eating disorder. Since BED is the most common ED, the results can be expected to also reflect association with BED. This limitation should be born in mind as we interpret the findings from different studies using DSM-V criteria.

Over70% of individual with different types of ED including BED reported co-morbidity disorders including anxiety (>40%), substance use (>10%), somatic symptoms; increased risk of suicide: risk factors included: parental psychiatric disorder, prenatal maternal stress, various family factors, childhood overweight and body dissatisfaction in adolescence. It was also found that only about one third of BED was detected by healthcare providers [6].

#### Why this study in Kenya

From the above literature review, various studies (nearly all of them from HIC) have reported various prevalence rates of BED ranging from less than 1%-4% [7, 8] and 5.6%-6.9% [9, 10] depending on country and study population. There is a dearth of literature on eating disorders not only in Kenya but the rest of Sub-Saharan Africa. Most of the countries in Sub-Saharan Africa are classified by the World Bank as Low and Middle Income Countries (LMIC ) [11]. However, there are improvements in incomes, standards of living, increased availability of food and an increase of outlets of fast foods especially in urban areas where 30% of the population live [12]. With the attendant increase in obesity and all its complications, eating disorders can no longer be ignored at clinical level and as a public health concern. Also, there is considerable evidence that epidemiological patterns of mental disorders in LMICs are similar to those in High Income Countries (HIC ) [13, 14]. Therefore, co-morbidity of eating disorders with mental disorders can be expected. So far, we have no Sub-Saharan Africa data that addresses BED, mental disorders and substance abuse as conditions that can co-morbid. This data is necessary to inform policy and practice on this co-morbidity.

### The goal and aims of our study

The overall objective of this study is to fill the knowledge gap in the prevalence of BED and associated co-morbidities in non-clinical populations of Kenyan students, and to generate data to inform clinical and public awareness.

**The specific aims are**: 1) To determine the socio-demographic and economic patterns associated with BED. 2) To document the prevalence of BED and prevalence of different symptoms of BED in a student population. 3) To determine associated psychiatric and substance use disorders and high risk for psychotic and affective disorders. 4) To determine independent predictors of BED.5) To use the findings of the study to suggest a strategy for a public health intervention that is context appropriate.

## Methods

### Recruitment and data collection

The study was a non-clinical, population-based cross-sectional study. Permission was sought from Institutional heads of the university and college students. For school going children in the community, permission was sought from the local administration (the schools were closed). This study was leveraged on an ongoing study on early psychosis [15–18] in the counties included in this study. However as explained, colleges and universities in Kenya admit students from all over the country some from rural schools and others from urban schools, and therefore represent a mix of these settings. We sent out an invitation through the community administration to parents with high school students for them to give permission for their children to participate in the study, and if agreeable to allow them to go to the data collection points. We have no information on the total number of students who were reached through this means of invitation. Some of the students came forward to participate in the study and for whom we have the numbers. All that we have is the total number that showed up, all of whom assented to participate in the study. Participants were recruited from Nairobi (Kenya’s capital) and three other counties in South Eastern Kenya - namely Machakos, Kitui and Makueni Counties. University and college students were approached after lecture hours in their classrooms. The research assistants were informed on the schedule for the different classes as they appeared in the timetables each day of the week. High school students were directed to specific public meeting areas for assessment with the help of the local community leaders. Participants were only included in the study if they met the requirements i.e. were in high school, college or university and had voluntarily agreed to participate in the study. We sought consent from participants aged 18+ and from parents of participants that were below the age of 18whoassented.The age range for high school students is 14-18 years. For college/university, the age range starts from 19-25 for most students but there are also late entry students. High school, college and university students in Kenya are all fluent in English – English is a national language and the official language for all communications and medium of learning. All participants, regardless of their age and provided they had been officially registered as students, were included and treated as students. A self-administered questionnaire was used to collect data from participants. A total of 9,742 participants from different years of study and courses were recruited for the study. As part of preparation for this study, we discussed with institutions on the need to incorporate mental health in their institutional health services for their students in case the awareness of mental disorders during the study prompted students to seek for mental health services. As for the high school students, we had trained staff at the local health facility on the World Health Organization (WHO) mental health GAP-Intervention Guide (mhGAP-IG). The WHO mhGAP-IG is a tool that was developed by World Health Organization for use in LMICs by trained lay health providers and training non-mental health specialists to provide mental health awareness on common mental disorders, how to provide first-aid and make referrals [19]. It has been used extensively in LMICs and its efficacy demonstrated by a recent literature and meta-analysis study [20], and more recently in Kenya by our team [19]. We also informed and directed the participants where to seek help in their institutions and community levels in case they needed any help. All the participants who were approached participated. This 100% response rate is not unique and is common in Kenyan community-based mental health related and student surveys [21, 22]. Students and parents place immense value on such surveys, as education is regarded as the best investment, with the highest potential to propel students into successful futures and help them and their families escape from poverty. However, there is need to point out that we approached colleges and university students at specific time points as a group, which may have contributed to the high response rate. The high school students voluntarily came to the community centers, indicating that they were already motivated to participate.

### Tools and measurements

#### BED

We used the Psychiatric Diagnostic Screening Questionnaire (PDSQ) which records BED symptoms based on DSM-IV.BED subscale of PDSQ showed good to excellent internal consistency in a study that involved994 psychiatric patients [23]. It is self-administered. PDSQ has 10 questions on BED each question asking about specific symptoms of BED. The questions were coded as No or Yes with ‘No’ having a value of zero and ‘Yes’ having a value of one.

#### Socio-demographic characteristics

A researcher-designed questionnaire was used to get the socio-demographics information of the respondents. Socio-demographic variables included age, gender, highest level of education, marital status and birth order.

#### Economic indicators

The respondents completed questions regarding household items, water source, toilet type and cooking method. These were used to estimate socio-economic status by creation of wealth index [24]. The wealth index used is based on the World Bank Recommendation for LMICs [24] and has been adopted by the Kenya Government for use in Kenya. It is classified into five sections; quintile 1-5 with quintile 1 representing the lowest level of wealth and 5 the highest level.

#### Psychiatric disorders

The psychiatric diagnostic screening questionnaire (PDSQ) was used to assess psychiatric conditions of the respondents. It consists of 126 questions assessing the symptoms of 13 *DSM-IV* Axis 1 disorders: mood disorders (major depressive disorder [MDD]); anxiety disorders (panic disorder, agoraphobia, PTSD, obsessive-compulsive disorder, generalized anxiety disorder [GAD], and social phobia); substance use disorders (alcohol abuse/dependence and drug abuse/dependence); and somatoform disorders (somatization disorder and hypochondriasis). In addition, it has a 6-item psychosis screen. For each psychiatric disorder, there are several questions which are computed to arrive at a diagnosis. The disorders chosen for coverage were selected because they are the most prevalent in epidemiological surveys of the community [25, 26] and the most frequently reported in large clinical samples [27–29]. In a validity study in which 994 psychiatric outpatients completed the scale [23], the 13 PDSQ subscales demonstrated good to excellent levels of internal consistency. Cronbach’s α was greater than .80 for 12 of the 13 subscales, and the mean of the α coefficients was .86. Test-retest reliability was examined in the 185 subjects who completed the PDSQ 2 times less than a week apart. Test-retest reliability coefficients were greater than 0.80 for 9 subscales, and the mean of the test-retest correlation coefficients was 0.83. The convergent and discriminant validity of the PDSQ subscales [30] was examined in 361 patients who completed a package of questionnaires at home less than a week after completing the PDSQ. The last six questions from PDSQ on major depressive episode domain are used to measure suicidal ideation classified as follows: frequently thinking of dying in passive ways like going to sleep and not waking up, wishing to be dead, thinking you were better off dead, having thoughts of suicide, seriously considering taking life, and thinking about specific ways of taking your life. The questions were coded as ‘No’ or ‘Yes’ with ‘No’ having a value of zero and ‘Yes’ having a value of one.

#### High risk for psychosis, affectivity and stress

The Washington Early Recognition Center Affectivity and Psychosis (WERCAP) screen was used to quantitatively assess psychosis-risk symptoms and bipolar-risk symptoms (affectivity) based on the frequency of symptoms and their effects on functioning [31]. It has high test-retest reliability and validity, with affectivity of sensitivity of .91, specificity of .71, psychosis sensitivity .88 and specificity of .8 2 [31].WERCAP has been validated for young people in USA [31], in Rwanda [32] and Kenya [15].

#### Drug and substance

The WHO’s Alcohol, Smoking, and Substance Involvement Screening Test (ASSIST) [33] was used only to determine the prevalence of different types of substance use on a ‘Yes’ or ‘No’ dimension. The WHO ASSIST has been used in Kenya [14] and other countries in Africa [34].

PDSQ symptoms are DSM-IV based. The diagnosis of BED, like all other DSM based diagnoses, is based on a configuration of symptoms with given duration and other behavioral considerations i.e. it is not based on the sum of symptoms which could be more in prevalence than the prevalence of BED as a diagnosis. Both DSM-IV and DSM-V are used globally in both HICs and LMICs for clinical and population studies. However, before we used PDSQ, we adapted it using a psychiatrist and clinical psychologists and found all the questions contextually appropriate and retaining the meaning in the DSM criteria. This is a common practice of adopting instruments developed and validated in one context and then applied in a different context [35, 36]. There was therefore no need to rephrase them.

### Data management and Analysis

The coded data was checked, cleaned and exported into Statistical Package for the Social Sciences (SPSS) version 23.0 for analysis. Basic descriptive statistics were carried out to estimate the prevalence of BED as well as the participant’s socio-demographic and socio-economic characteristics. Scores were grouped into two: those with BED and those without BED. Estimation of univariate associations between BED and other variables was carried out by fitting bivariate logistic regression that was also used to identify confounding factors. Variables with *p*-value less than 0.05 were then fitted into generalized linear model, with logit as the link, to identify independent predictors of BED. The strength and significance of the association between the variables and BED was assessed by calculating the adjusted odds ratio with a 95% confidence level. Correlation analysis was also carried out between BED and the psychiatric conditions. All the tests carried out were two-sided with a set *P*-value of less than 0.05 (*p*<0.05).

## Results

### The results are arranged in a sequence that reflects the sequence of the stated aims

Table 1 presents the results of socio-demographic characteristics of the respondents. Males (53.5%) were more than females. Mean age was 21.4, median 21.3 (range 15-43) years. Majority of the respondents were single (93.4%), mainly from university (68.6%), most (56.9%) were either first or second born in their families. The wealth index was evenly distributed among the first 5 quintiles with the fifth quintile having the lowest proportion (16.6%).

**Table 1:** Socio-demographic and economic factors of respondents

Variable	Category	Frequency(*N*=9742)	Percentage (%)
Gender	Male	5173	53.5
	Female	4500	46.5
	*Missing*	*69*	*0.7*
Age	Mean; Median; SD; Range	21.4; 21.3; 2.4; 15-43	
Marital status	Married	607	6.3
	Single	9057	93.4
	Others	38	0.4
	*Missing*	*40*	*0.4*
Religion	Protestant	5512	57.1
	Catholic	3359	34.8
	Muslim	410	4.2
	Other	368	3.8
	*Missing*	*93*	*1.0*
Birth order	1-2	5539	56.9
	3-5	3271	33.6
	6+	920	9.5
	*Missing*	*12*	*0.1*
Level of Education^a^	High School	1506	15.5
	College	1534	15.8
	University	6648	68.6
	*Missing*	*54*	*0.6*
Wealth Index (*Quintile1=Lowest;* *Quintile 5=Highest)*	Quintile 1	2044	21.0
	Quintile 2	1865	19.1
	Quintile 3	2002	20.6
	Quintile 4	2214	22.7
	Quintile 5	1617	16.6

^a^In Kenya, high school students are aged 14-18 while college and university students are aged 18+

Table 2 summarizes the socio-demographic factors associated with BED at bivariate level. The younger age group, representing high school students were 1.39 times more likely to have BED as compared to those in the university (*p*<0.05). Level of education was the only socio-demographic factor that was associated with BED.

**Table 2:** Socio-demographic factors associated with binge  eating  disorder

Variable	Category	Binge Eating Disorder		O.R (95% C.I.)	Sig.
		No	Yes		
Gender	Male	5005(96.8%)	167(3.2%)	1.00(0.80-1.26)	0.987
	Female	4354(96.8%)	145(3.2%)	Ref.	
Age	Mean±SD;	21.4±2.4	21.1±2.5	1.01(0.97-1.06)	0.619
Marital status	Married	589(97.0%)	18(3.0%)	0.36(0.10-1.27)	0.111
	Single	8765(96.8%)	290(3.2%)	0.39(0.12-1.26)	0.115
	Others	35(92.1%)	3(7.9%)	Ref.	
Religion	Protestant	5353(97.1%)	158(2.9%)	0.96(0.52-1.78)	0.892
	Catholic	3223(96.0%)	135(4.0%)	1.36(0.73-2.54)	0.335
	Muslim	401(97.8%)	9(2.2%)	0.73(0.30-1.78)	0.486
	Other	357(97.0%)	11(3.0%)	Ref.	
Birth order	1-2	5343(96.5%)	194(3.5%)	1.12(0.75-1.66)	0.589
	3-5	3178(97.2%)	93(2.8%)	0.90(0.59-1.37)	0.622
	6+	891(96.8%)	29(3.2%)	Ref.	
Level of Education^a^	High School	1442(95.8%)	63(4.2%)	1.39(1.04-1.85)	**0.026**
	College	1487(96.9%)	47(3.1%)	1.00(0.73-1.38)	0.984
	University	6444(96.9%)	203(3.1%)	Ref.	
Wealth Index	Quintile 1	1891(97.3%)	53(2.7%)	0.82(0.56-1.18)	0.282
	Quintile 2	1886(97.1%)	57(2.9%)	0.88(0.61-1.27)	0.491
	Quintile 3	1833(96.4%)	69(3.6%)	1.10(0.78-1.55)	0.602
	Quintile 4	1879(96.4%)	71(3.6%)	1.10(0.78-1.55)	0.585
	Quintile 5	1864(96.7%)	64(3.3%)	Ref.	

*Ref* Reference Category, *C.I* Confidence Interval; *O.R* Odds Ratio

^a^In Kenya, high school students are aged 14-18 while college and university students are aged 18+. Statistical model: Logistic regression model; covariate-age

**Fig. 1:**
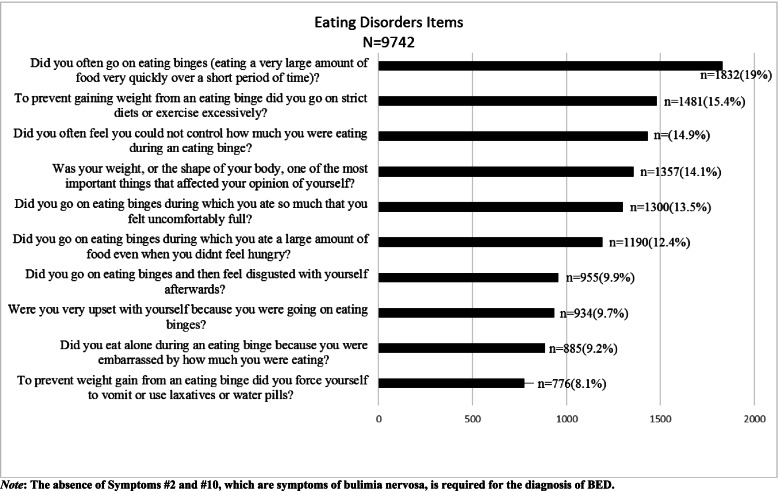
Prevalence of Binge Eating Disorder and Related Symptoms. *Note*: The absence of Symptoms #2 and #10, which are symptoms of bulimia nervosa, is required for the diagnosis of BED

The overall prevalence of BED was 3.2%. Figure 1 presents a pictorial representation in descending order of BED and BED related symptoms using 10 PDSQ items used to screen for BED. The symptom on often going on eating binges (19.0%) was highly prevalentwhile the symptom on vomiting and use of laxative or water pills as a way to prevent weight gain (8.1%) was least prevalent.

Table 3 presents the results on the psychiatric disorders associated with BED at bivariate level. There was significant association between all psychiatric disorders and BED (*p*<0.001). Major depressive disorder (11.0%) was the most common while OCD (4.7%) was the least common psychiatric disorder.

**Table 3:** Psychiatric Disorders associated with BED

Condition	Category	BED		O.R (95% C.I.)	Sig.
		No	Yes		
Major Depressive Disorder:	No	7608(98.8%)	92(1.2%)	Ref.	
	Yes	1816(89.0%)	224(11.0%)	10.20(7.96-13.07)	**<0.001**
PTSD:	No	6973(98.4%)	115(1.6%)	Ref.	
	Yes	2451(92.4%)	201(7.6%)	4.97(3.94-6.28)	**<0.001**
Obsessive Compulsive Disorder:	No	3411(99.4%)	21(0.6%)	Ref.	
	Yes	6013(95.3%)	295(4.7%)	7.97(5.11-12.43)	**<0.001**
Panic Disorder:	No	7735(98.6%)	110(1.4%)	Ref.	
	Yes	1688(89.1%)	206(10.9%)	8.58(6.77-10.88)	**<0.001**
Psychosis:	No	5644(99.0%)	56(1.0%)	Ref.	
	Yes	3780(93.6%)	260(6.4%)	6.93(5.18-9.28)	**<0.001**
Agoraphobia:	No	6321(98.9%)	68(1.1%)	Ref.	
	Yes	3102(92.6%)	248(7.4%)	7.43(5.66-9.75)	**<0.001**
Social Phobia:	No	4802(98.9%)	54(1.1%)	Ref.	
	Yes	4622(94.6%)	262(5.4%)	4.70(3.74-5.90)	**<0.001**
Alcohol Abuse/Dependence:	No	7395(98.1%)	147(1.9%)	Ref.	
	Yes	2029(92.3%)	169(7.7%)	4.19(3.34-5.25)	**<0.001**
Drug Abuse/Dependence:	No	7904(97.9%)	166(2.1%)	Ref.	
	Yes	1520(91.0%)	150(9.0%)	5.90(4.69-7.43)	**<0.001**
Generalized Anxiety Disorder:	No	8266(97.9%)	173(2.1%)	Ref.	
	Yes	1158(89.0%)	143(11.0%)	4.27(3.40-5.36)	**<0.001**
Somatization Disorder:	No	7104(98.2%)	132(1.8%)	Ref.	
	Yes	2320(92.7%)	184(7.3%)	10.20(7.96-13.07)	**<0.001**
Hypochondriasis:	No	6972(98.3%)	122(1.7%)	Ref.	
	Yes	2452(92.7%)	194(7.3%)	4.52(3.59-5.70)	**<0.001**
Suicidality	No	7416(98.4%)	120(1.6%)	Ref.	
	Yes	2008(91.1%)	196(8.9%)	6.03(4.78-7.61)	**<0.001**
Sum Score of WERC Stress Screen:	Mean±SD;	25.2±26.4	49.9±38.4	1.02(1.02-1.02)	**<0.001**
Total sum of WERCAP Affectivity	Mean±SD;	10.2±8.3	17.2±9.8	1.09(1.07-1.10)	**<0.001**
Total sum of WERCAP Psychosis:	Mean±SD;	8.4±9.6	18.2±13.1	1.07(1.06-1.08)	**<0.001**

*Ref* Reference Category, *C.I* Confidence Interval, *O.R* Odds Ratio. Statistical model; Logistic regression model, covariates-WERCAP Affectivity and Psychosis, *WERC* Stress screen

The correlation between BED and psychiatric disorders was highly significant (*p*<0.001) for all psychiatric disorders (Table 4).

**Table 4:** Correlation between binge  eating disorder scores and psychiatric conditions scores

Pearson Correlation	1	2	3	4	5	6	7	8	9	10	11	12	13	14	15	16
1. Binge Eating Disorder	1															
2. Major Depressive Disorder Score:	.488^**^	1														
3. PTSD Score:	.412^**^	.514^**^	1													
4. Obsessive Compulsive Disorder.	.377^**^	.426^**^	.391^**^	1												
5. Panic Disorder Score:	.442^**^	.497^**^	.449^**^	.526^**^	1											
6. Psychosis Score:	.466^**^	.467^**^	.443^**^	.476^**^	.563^**^	1										
7. Agoraphobia Score:	.396^**^	.432^**^	.389^**^	.461^**^	.502^**^	.472^**^	1									
8. Social Phobia Score:	.363^**^	.462^**^	.376^**^	.469^**^	.453^**^	.425^**^	.573^**^	1								
9. Alcohol Abuse/Dependence	.380^**^	.316^**^	.269^**^	.211^**^	.298^**^	.334^**^	.284^**^	.275^**^	1							
10. Drug Abuse/Dependence	.371^**^	.295^**^	.267^**^	.199^**^	.258^**^	.312^**^	.247^**^	.233^**^	.617^**^	1						
11. Generalized Anxiety Disorder Score:	.376^**^	.519^**^	.413^**^	.425^**^	.486^**^	.424^**^	.468^**^	.556^**^	.275^**^	.276^**^	1					
12. Somatization Disorder Score:	.344^**^	.411^**^	.333^**^	.301^**^	.391^**^	.354^**^	.377^**^	.377^**^	.298^**^	.290^**^	.424^**^	1				
13. Hypochondriasis Score:	.350^**^	.397^**^	.339^**^	.304^**^	.424^**^	.383^**^	.376^**^	.365^**^	.305^**^	.314^**^	.451^**^	.525^**^	1			
14. WERC Stress Screen score.	.308^**^	.438^**^	.362^**^	.311^**^	.339^**^	.299^**^	.300^**^	.311^**^	.217^**^	.211^**^	.345^**^	.275^**^	.280^**^	1		
15. Total sum of WERCAP Affectivity Disorder:	.302^**^	.504^**^	.348^**^	.322^**^	.338^**^	.329^**^	.303^**^	.334^**^	.183^**^	.178^**^	.409^**^	.294^**^	.276^**^	.415^**^	1	
16. Total sum of WERCAP Psychosis:	.353^**^	.487^**^	.382^**^	.353^**^	.393^**^	.445^**^	.339^**^	.327^**^	.242^**^	.231^**^	.386^**^	.291^**^	.316^**^	.418^**^	.660^**^	1

*P<0.05; **P<0.01, ***P<0.001

Table 5 presents the results on drug/substances associated with BED at bivariate level. There was significant association between all the substances and BED except for alcohol (*p*<0.05). The prevalence of SUD in BED varied from 3.7% to 7.6%. Alcohol had the lowest prevalence of 3.7%.

**Table 5:** Drug/Substances associated with binge eating disorder

Substance	Category	Binge Eating Disorder Score:		O.R (95% C.I.)	Sig.
		No	Yes		
Tobacco	No	8930(96.9%)	287(3.1%)	Ref	
	Yes	494(94.5%)	29(5.5%)	0.55(0.37-0.81)	**0.003**
Alcohol	No	7768(96.9%)	252(3.1%)	Ref	
	Yes	1656(96.3%)	64(3.7%)	1.19(0.90-1.58)	0.219
Cannabis	No	8981(96.9%)	292(3.1%)	Ref	
	Yes	443(94.9%)	24(5.1%)	1.67(1.09-2.55)	**0.019**
Sedatives	No	9266(96.8%)	303(3.2%)	Ref	
	Yes	158(92.4%)	13(7.6%)	2.52(1.41-4.48)	**0.002**
Khat/Amphetamine	No	9151(96.8%)	298(3.2%)	Ref	
	Yes	273(93.8%)	18(6.2%)	2.02(1.24-3.31)	**0.005**

*Ref* Reference Category, *C.I* Confidence Interval, *O.R* Odds Ratio. Statistical model: Logistic regression model

Table 6 summarizes the independent predictors of BED using AOR analysis. Major depressive disorder, OCD, panic disorder, psychosis, agoraphobia, drug abuse, generalized anxiety disorder, suicidality and WERCAP stress were the leading independent predictors of BED.

**Table 6:** Independent Predictors of binge eating disorder

Category	A.O.R.	95% C.I A.O.R.		Sig.
		Lower	Upper	
Level of Education				
University	1.011	0.738	1.386	0.943
College	0.804	0.531	1.218	0.304
High School	Ref			
Tobacco				
Yes	0.986	0.605	1.606	0.955
No	Ref			
Cannabis				
Yes	0.906	0.527	1.560	0.723
No	Ref			
Sedatives				
Yes	1.059	0.547	2.048	0.866
No	Ref			
Khat/amphetamine				
Yes	1.231	0.691	2.192	0.481
No	Ref			
Major Depressive Disorder				
Yes	2.478	1.807	3.399	**<0.001**
No	Ref			
PTSD				
Yes	1.039	0.785	1.377	0.788
No	Ref			
Obsessive Compulsive Disorder				
Yes	1.810	1.105	2.965	**0.018**
No	Ref			
Panic Disorder				
Yes	2.059	1.548	2.740	**<0.001**
No	Ref			
'Psychosis				
Yes	1.406	0.988	2.000	0.058
No	Ref			
Agoraphobia				
Yes	1.959	1.409	2.724	**<0.001**
No	Ref			
Social Phobia				
Yes	0.879	0.612	1.263	0.487
No	Ref			
Alcohol Abuse/Dependence				
Yes	1.236	0.895	1.708	0.198
No	Ref			
Drug Abuse/Dependence				
Yes	1.552	1.122	2.147	**0.008**
No	Ref			
Generalized Anxiety Disorder				
Yes	1.681	1.277	2.213	**<0.001**
No	Ref			
Somatization Disorder				
Yes	1.216	0.925	1.598	0.161
No	Ref			
Hypochondriasis				
Yes	0.891	0.664	1.195	0.442
No	Ref			
Suicidality				
Yes	1.639	1.243	2.161	**<0.001**
No	Ref			
Total sum of WERCAP Affectivity:	1.004	0.986	1.022	0.660
Total sum of WERCAP Psychosis:	1.009	0.996	1.023	0.177
Sum Score of WERC Stress Screen:	1.005	1.001	1.008	**0.022**

*Ref* Reference Category, *C.I* Confidence Interval, *n/s* Not significant, *A.O.R* Adjusted Odds Ratio. Statistical model: Generalized linear model; covariates-WERCAP Affectivity and Psychosis, *WERC* Stress screen

## Discussion

To the best of our knowledge, our study is unique in two ways: first African study of its kind and one of the two reported globally on college and university students (the other one was done in USA college students). Additionally, our study includes high school students. Our large sample study population provides valuable comparison with similar large population samples in HIC reported in the Introduction.

### Prevalence of BED and BED related symptoms

The overall prevalence of BED (3.2%)found in our study is much higher than the “estimated” general population lifetime prevalence of BED of 0.3-1.6% reported by Swanson et al [37], but within the range of 0.7– 4.3% reported by American Psychiatric Association (APA) [38]. These similar findings from different socio-cultural contexts could be taken to mean culture has no influence on prevalence of BED. Even much higher in our study are the prevalence rates of individual BED symptoms. We cannot generalize findings on high school student population to other similar students because our study participants came from a specific community. However, the college and university students who participated in this study are drawn from across the country and therefore there is a possibility that our findings can be generalized to college and university students admitted under the same arrangements to other colleges and universities in Kenya. Further, given the pyramid structure of the Kenyan population [12], these students represent a significant proportion (45.7%) [39] of the total Kenyan population, that is, our findings represent nearly half of the Kenyan population. This is more relevant given that there is growing evidence that BED affects all sections of the society [40]. It is noteworthy that our findings coming from an LMIC setting, often associated with limited resources and often reported malnutrition or shortage of food [41], are similar to those reported by American Psychiatric Association. The prevalence of the different symptoms in our study (19.0% to 8.1%) has relevance to creating awareness both in public and clinical settings. Recognition of any one of them could provide the cue to enquire for more, leading to a possible diagnosis of BED.

### The Socio-demographics

The finding that males were more than the females could be explained in several ways: Firstly, most of the college students were in technical colleges that are patronized by male than female gender; and secondly, the data collection for high school students was done when the schools were closed in a cultural context where boys more than girls were more likely to be allowed by their parents to go to the data collection site. The wide range in age is easily explained by late entry into schools, colleges and university. Since most of the participants were students, it is not surprising that most were single. Other types of marriages were come-we-stay arrangement common in college and university students as one of the ways of sharing accommodation costs. Kenyan family structure is increasingly getting smaller, explaining 1^st^ and 2^nd^ birth order [12].

### Co-morbidity of BED with psychiatric disorders and SUD

Our study findings agree with the global trends (i.e. other population studies in HICs referenced in the Introduction), that BED is associated with various psychiatric disorders. However, our study did not explore the cause-effect relationship between different psychiatric disorders and BED. Furthermore, our findings agree with global trends that there is an association between BED and substance use disorder (SUD). However, we found an average of 3.1% to 7.6% association with SUD, which is lower than the lifetime reported prevalence of 30% to 70% lifetime use in BED reported elsewhere in HICs [42, 43] referenced in the Introduction. These differences could be attributed to different methodologies of collecting data on SUD or indeed different patterns of substance use in different contexts. We did not however study the nature of association between BED and SUD–whether causal or casual. Qualitative and biomarker studies are required to elucidate the nature of this association even in the Kenyan setting. These findings of prevalence and associated disorders call for awareness campaigns in the Kenyan situation similar to what has been suggested in HICs [44–46].

### Independent predictors

It is noteworthy that no socio-demographic variables or wealth indicators were independent predictors of BED, suggesting that BED is not a condition necessarily associated with affluence, at least in our study participants. Most of the independent predictors were major depression and various types of anxiety disorders and increased stress levels. It is therefore not surprising that sedatives that are essentially anxiolytics were significantly associated with BED. Though both alcohol disorder and SUD were more in BED than in Non-ED, it was SUD that achieved significant levels as an independent predictor of BED.

### Implications of our findings

There is need for awareness campaigns on BED and voluntary screening for BED especially in patients with psychiatric disorders and SUD. Awareness campaigns should target the general population on the various symptoms of BED and the potential co-morbidity of psychiatric disorder and/or substance use disorders (SUD). This could take place in the form of general health education using the social media which has become the most powerful influential and cost-effective means of communication. The purpose is to create awareness for people who have BED and do not know. It will also reach out to people who do not have BED but know other people (friends, family members etc) in whom they see symptoms of BED. Health service providers should also be targeted for awareness so that they can be sensitive to the possibility of BED in their patients, especially if there is obesity and associated mental disorders or substance use disorders. These can be effected through inclusion of eating disorders in their training curriculums and during continued education programs. The increased awareness could lead to voluntary screening using the DSM-IV criteria for confirmation of suspected BED followed by health seeking behaviour and hopefully management.

### Future Studies

Mixed methods i.e. qualitative and quantitative including biomarkers and a longitudinal approach would determine the nature of the associations.

### Strengths of the study

A large sample of 9742representingan age group that constitutes nearly 50% of Kenyan population. 

#### Limitations of the study

(i) Cross-sectional quantitative only research design.(ii) There were late age entry students but the contribution of age was determined at analysis level and found that age was not a predictive factor .iii) The overall effect is that we can only talk of "probable" BED because BED symptoms were assessed by self-report and self-report assessment of these symptoms is known to be highly unreliable. In mitigation, this would apply to any other studies reported in the literature done using the same method i.e. self-screening which found prevalence similar to our finding. iv) PDSQ has not been validated for this study population. The mitigation for this has already been expounded under Methods.

## Conclusion

We have achieved prima facie evidence for the following in our study participants: 1) There is an association between BED and socio-economic factors, 2) BED and its various symptoms are prevalent, 3) different psychiatric disorders and SUD co-morbid with BED, 4) there are independent predictors of BED and 5) there is need for awareness campaigns of BED.

Thus, we have provided new information on the global data on BED. We recommend prospective mixed methods future studies.

## Data Availability

The data-sets used and/or analysed during the current study available from the corresponding author on reasonable request.
